# Descriptive Epidemiology of *Klebsiella* spp. Urinary Tract Infections in Central Africa

**DOI:** 10.1155/bmri/9558259

**Published:** 2025-12-21

**Authors:** Evrard Mayombo Ngoussou, Franck Mounioko, Mambu Mundunge, Rolande Mabika Mabika, Ornella Zong Minko, Léonce Fauster Ondjiangui, Jean Fabrice Yala

**Affiliations:** ^1^ Bacteriology Laboratory, Medical Analysis Research Unit, Interdisciplinary Center of Medical Research of Franceville (CIRMF), Franceville, Gabon; ^2^ Vector Systems Ecology Unit, Interdisciplinary Center of Medical Research of Franceville (CIRMF), Franceville, Gabon; ^3^ National Public Health Laboratory, Libreville, Gabon, nphl.gov.np; ^4^ Molecular and Cellular Biology Laboratory, Microbiology Team (LABMC), Agrobiology Research Unit, Masuku University of Sciences and Techniques (USTM), Franceville, Gabon

## Abstract

**Objective:**

Urinary tract infections are the most common bacterial infections encountered in clinical practice. Among the pathogens responsible, bacteria of the *Klebsiella* spp. are the second most frequently isolated uropathogenic agents worldwide. These bacteria are constantly evolving, both epidemiologically and in terms of the development of antimicrobial resistance. In Central Africa, available data on the spread of *Klebsiella* spp. are mainly derived from isolated studies, making it difficult to obtain an overview of their epidemiology in the subregion. Consequently, these systematic review and meta‐analysis are aimed at estimating the pooled prevalence of urinary tract infections in Central Africa and to describe the epidemiology of the *Klebsiella* spp. strains responsible for these infections.

**Methods:**

Relevant articles were searched in the SCOPUS, PubMed, and Google Scholar databases. The study selection process was conducted in accordance with the PRISMA flowchart recommendations. Systematic review and meta‐analysis were used to summarize data on urinary tract infections. Prevalence was determined and visualized using a forest plot with R software Version 4.4.1. Also, finally, geographical mapping of the data distribution was carried out using QGIS software (Version 3.34.15‐Prizren).

**Result:**

Out of all the articles retrieved, 34 studies were deemed eligible for this analysis. The overall pooled prevalence of urinary tract infections in Central Africa was estimated at 28% (95% IC: 28, 29). The overall isolation rate of *Klebsiella* spp. responsible for urinary tract infections was 12% (95% IC: 11, 12). Analysis of the distribution of *Klebsiella* spp. isolation rates in urinary tract infections across Central Africa revealed variability by country, ranging from 10% to 25%. The species *Klebsiella pneumoniae* was the most frequently isolated, present in 96.15% of the studies. Furthermore, *Klebsiella* spp. strains responsible for urinary tract infections were predominantly identified in females, with an overall isolation rate of 82.23%, compared to 17.77% in males.

## 1. Introduction

In human clinical practice, urinary tract infections (UTIs) are the most frequently encountered infections, both in the general population and in hospitals [1]. These infections are a public health problem for many patients in different parts of the world, with high medical costs. Studies carried out in Germany, Peru in women, and Africa recorded UTI prevalences of 24.60%, 27.60%, and 43.28%, respectively [2–4]. These infections affect men and women of all ages, with a much higher incidence in women than in men. Indeed, UTIs can have significant consequences, especially in pregnant women. It is well established that these infections can be associated with complications such as intrauterine growth retardation and low birth weight. This underlines the importance of careful medical monitoring during pregnancy to detect and promptly treat any UTI [5]. In children, they also cause additional long‐term kidney damage [6]. Worldwide, a 60.40% increase in cases of UTIs was recorded between 1990 and 2019, as well as a 2.4‐fold increase in deaths attributed to these infections over the same period [7]. It is worrying to see an increase in this type of infection on such a scale. This sharp rise in UTIs underlines the importance of raising awareness and preventing them. UTIs are particularly prevalent in sub‐Saharan Africa, where access to high‐quality care and advanced bacteriological diagnostic tools iscomplicated. In addition, rudimentary and inadequate hygiene conditions in some households contribute to the spread and increase of these infections [8, 9]. This underlines the importance of improving sanitary infrastructures and educating people about hygiene practices. This trend is in fact correlated with studies on UTIs worldwide, which reveal that the highest incidence of UTIs was reported in the African Region, which accounted for 3.60, while the lowest incidence was reported in the Western Pacific Region with 0.40, followed by the Eastern Mediterranean Region with 1.10 and finally the American Region with 1.90 [10].

UTIs are highly diverse both etiologically and clinically. They can be caused by different types of bacteria, and their symptoms can vary considerably from one person to another. Some UTIs may manifest themselves with mild symptoms, such as pain or burning on urination, while others may progress to more serious forms, leading to serious complications if not treated promptly. UTIs can be bacterial, viral, or even fungal [11]. Bacterial adhesion to uroepithelial cells is essential for the initiation of UTI, making Gram‐negative bacteria, particularly those belonging to the Enterobacterales family, the main uropathogens [10]. *Escherichia coli* is the main pathogen responsible for UTIs, followed by *Klebsiella* spp. Indeed, bacteria of the *Klebsiella* genus have been reported in numerous studies to be the second most common uropathogen. Studies in Ethiopia and America show that *Klebsiella* spp. uropathogen was responsible for 22.20% and 6.80% of UTIs, respectively [12–14].

Bacteria of the *Klebsiella* genus are Gram‐negative bacilli found everywhere in the environment, but also in humans, where they often colonize mucous surfaces, the upper respiratory tract, and the intestine. However, colonization rates of these bacteria vary considerably from one individual to another, depending on their habitat and exposure [15, 16]. *Klebsiella pneumoniae*, *Klebsiella oxytoca*, and *Klebsiella aerogenes* (formerly *Enterobacter aerogenes*) in particular are the focus of most clinical attention [17]. *Klebsiella pneumoniae* has been classified by the WHO as one of the ESKAPE organisms (*Enterococcus faecium, Staphylococcus aureus, Klebsiella pneumoniae, Acinetobacter baumannii, Pseudomonas aeruginosa*, and *Enterobacter*), which are well‐known clinical pathogens with increasing multidrug resistance and virulence [18]. Bacteria of the *Klebsiella* genus are known for their ability to cause infections due to several virulence factors, the most important of which are the capsule, fimbriae, lipopolysaccharide, and siderophores [19]. Also, numerous studies suggest the rise of *Klebsiella* spp. as a human pathogen, especially with its increasing prevalence in human infections and antimicrobial resistance. This makes the treatment of infections increasingly complex and raises major challenges for healthcare professionals. Antimicrobial resistance is a global public health problem that leads to complications in the treatment of infections, increasing the risk of death [15]. This situation calls for increased attention and research efforts to develop new treatments and prevention strategies. Practitioners and researchers need to work together to find innovative solutions, whether through the development of new antibiotics, the use of alternative therapies, or improved infection prevention practices.

It is true that the lack of data on the epidemiology of UTIs in Africa in general, and Central Africa in particular, poses a significant challenge for public health. Understanding the distribution and involvement of bacteria, such as those of the *Klebsiella* genus, is crucial to developing effective prevention and treatment strategies. Without this information, it is difficult to assess the scale of the problem and tailor the necessary interventions. It would therefore be beneficial to carry out in‐depth studies to gather reliable data on this subject, which could help improve the management of UTIs in this region. The present literature review was motivated by the need to deepen our understanding of the epidemiology of UTIs caused by *Klebsiella* spp. bacteria in Central Africa. Indeed, UTIs represent a major public health problem in this region, and it is crucial to have accurate, up‐to‐date data to better target interventions. Thus, the main objective was to determine the overall prevalence of UTIs in Central Africa, and specifically, the rate of infections attributed to *Klebsiella* spp. In addition, this study was aimed at analyzing the distribution of these infections according to different criteria such as country, year, gender, and bacterial species. This will not only provide a better understanding of the current situation but also guide future research and public health strategies.

## 2. Material and Methods

The PRISMA (Preferred Reporting Items for Systematic Reviews and Meta‐Analysis) guideline was used to carry out this systematic review. This systematic review was to provide an epidemiological description of *Klebsiella* spp. strains implicated in UTIs in Central Africa. It is registered on PROSPERO under the number CRD420245572022 and available at the following web address: http://crd.york.ac.uk/prospero/display_record.php?RecordID=572022.

### 2.1. Information Sources and Search Strategy

A systematic search was conducted in the PubMed, Scopus, and Google Scholar databases, covering the period from 2000 to 2024. The search strings combined terms related to UTIs, the bacterium *Klebsiella* spp., and the targeted geographical region (Central Africa). The following Boolean operators were used to refine the results: (“urinary tract infection” OR “urinary infection” OR “urinary tract infections”) AND (“*Klebsiella*” OR “*Klebsiella* spp” OR “uropathogen”) AND (“Central Africa” OR “Cameroon” OR “Gabon” OR “Republic of Congo” OR “Central African Republic” OR “Equatorial Guinea” OR “Angola” OR “ Sao Tome and Principe” OR “Chad” OR “Democratic Republic of Congo”).

#### 2.1.1. Inclusion Criteria

Studies that met the following inclusion criteria were included in the systematic review:

• Study population: The study population was made up of patients of all genders and ages admitted to healthcare establishments for bacteriological examination of urine.
• Results: They include all studies reporting quantitative results, that is, the size of the study population, the number of UTIS‐positive patients, the frequency, rate or incidence of UTIs, and the number or rate of isolation of *Klebsiella* spp. from patients.
• Language: Only articles written in English were included
• Types of articles: full text, original articles and peer‐reviewed published articles.
• Years of publication: articles published between 2000 and 2024.
• Regions or countries of study: Falling within the Central African region, comprising the following countries according to the geographical delimitation of the United Nations (UN), Angola, Cameroon, Gabon, Equatorial Guinea, Central African Republic (CAR), Democratic Republic of Congo (DRC), Republic of Congo (RC), Sao Tome and Principe, and Chad [20, 21].

#### 2.1.2. Exclusion Criteria

Studies that did not report quantitative results on the study population, the prevalence or incidence of UTIs, or the prevalence of *Klebsiella* spp. involved in these infections. Also excluded were case series, review articles, dissertation reports, conference abstracts, and unavailable articles.

### 2.2. Study Article Selection Process

The article selection process was carried out using the PRISMA flowchart, indicating the number of articles included and excluded from the study along with the reasons for exclusion. Following article searches in the included electronic databases, duplicate articles were removed using Mendeley Desktop Version 1.19.8.

The remaining articles were then independently reviewed according to their titles and abstracts to determine their eligibility by applying the inclusion criteria.

### 2.3. Data Extraction Process

Data extraction was performed independently by two trained reviewers to ensure the reliability and consistency of the information collected. Variables extracted included year of publication, country of origin, total number of participants, isolation rate of uropathogenic strains of *Klebsiella* spp., and diagnostic methods used. In the event of disagreement between the extractors, a discussion was initiated to reach a consensus, and, if necessary, a third researcher intervened as an arbitrator.

### 2.4. Statistical Data Analysis

A systematic review and meta‐analysis were used to summarize data on UTIs by pooling the results of studies reporting the prevalence of UTIs in Central Africa. The prevalence of these infections in the various studies and overall in Central Africa, as well as the isolation rate of uropathogenic *Klebsiella* spp. strains, were determined and visualized using a forest plot with R software Version 4.4.1. The Pearson test was used to compare variables, and significance was considered for a value of *p* ≤ 0.05. Geographical visualization of these data was carried out using QGIS software Version 3.34.15‐Prizren. For the meta‐analysis, a mixed‐effects model (random‐effects model) was used to account for variability between studies. Heterogeneity between studies was assessed using the *I* ^2^ index, which measures the percentage of total change attributable to heterogeneity rather than chance. *I* ^2^ values above 75% indicate high heterogeneity. Publication bias was examined by the construction of funnel plots and statistically tested using the Egger test to detect an asymmetry suggesting the presence of publication bias.

## 3. Results

### 3.1. Study Characteristics

**Figure 1:**
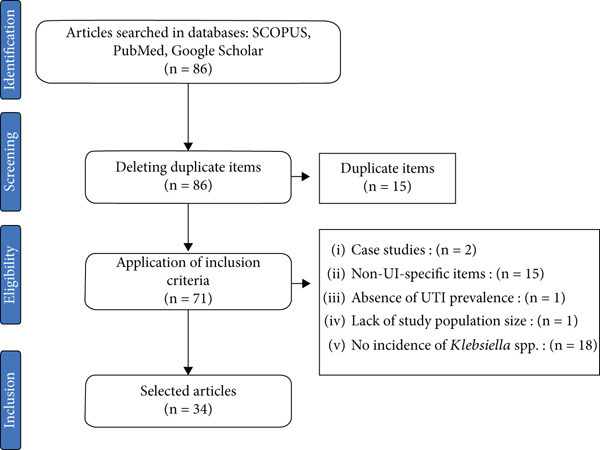
PRISMA diagram of the articles selected and included in the study.

The search for articles on PubMed, Scopus, and Google Scholar related to *Klebsiella* spp. strains involved in UTIs in Central African countries yielded a total of 86 articles. After scouring this body of works using the study′s inclusion criteria and deleting duplicate articles, 34 articles were selected for inclusion in this study (Figure 1).

Details related to the selected articles were recorded and included full references, study periods, countries and cities of origin, and more, as summarized in Table 1. From these studies, it transpired that the present review covers a total population of 31,158 patients of all sexes and ages. The analysis of Table 1 reveals that the studies took place between 1997 and 2023. These studies were carried out in the following countries: Cameroon (18), DRC (7), Gabon (3), Chad (2), RCA (1), Republic of Congo (2), and Equatorial Guinea (1). However, Angola and Sao Tomé et Principe recorded no studies.

**Table 1:** Characteristics of the different studies included.

**Authors**	**Study period**	**Country**	**Study city**	**Study population**	**Workforce**
(Yuyun et al., 2004) [22]	April 1996–October 1997	Cameroon	Yaoundé	Adult men	179
(Bercion et al., 2006) [23]	January 2004–December 2006	RCA	Bangui	Everyone	5128
(Njunda et al., 2012) [24]	March–September 2011	Cameroon	Buea et Limbe	Diabetic	125
(Akoachere et al., 2012) [25]	2012	Cameroon	Bamenda et Buea	Everyone	235
(Mokube et al., 2013) [26]	July 2012	Cameroon	Buea	Pregnant women	102
(Bissong et al., 2013) [27]	2013	Cameroon	Buea	Diabetic/nondiabetic patients	265
(Bayaba and Dimani, 2014) [28]	February–May 2023	Cameroon	Dschang et Bafoussam	Everyone Everyone	215
(Irenge et al., 2014) [29]	September 2012–August 2013	RDC	Bukavu	Everyone	2724
(Abderrazzack et al., 2015) [30]	January–March 2014	Chad	N’Djamena	Pregnant women HIV	76
(Chatalov, 2015) [31]	August 2013–September 2015	Equatorial Guinea	Malabo	Everyone	785
(Ndamason et al., 2016) [32]	December 2015–May 2016	Cameroon	Bamboutos Division	Pregnant women/nonpregnant	128
(Nzalie, Gonsu and Koulla‐shiro, 2016) [33]	January–April 2014	Cameroon	Yaoundé	Everyone	92
(Malou, Makoutode and Kaj, 2017) [34]	October 2014–April 2015	RDC	Lubumbashi	Women	207
(Bunduki, Kibendelwa and Nzanzu, 2017) [35]	January 2015–December 2016	RDC	Butembo	Everyone	617
(Nguefack et al., 2019) [36]	January–April 2015	Cameroon	Douala	Pregnant women	354
(Yandai et al., 2019) [37]	June 2014–December 2016	Chad	N’Djamena	Everyone	503
(Francine et al., 2019) [38]	May 2013–March 2014	Cameroon	Douala	Children	400
(Ebob et al., 2019) [39]	February–June 2016	Cameroon	Bamenda	Everyone	98
(Signing et al., 2020) [40]	August 2018–May 2019	Cameroon	Bafoussam	Diabetic/nondiabetic patients	505
(Nji et al., 2020) [41]	2020	Cameroon	Buea	Children	405
(Djim‐adjim‐ngana et al., 2020) [42]	June–September 2018	Cameroon	Garoua	Children	57
(Egbe et al., 2020) [43]	January–April 2019	Cameroon	Douala	Pregnant women	206
(Samje et al., 2020) [44]	2020	Cameroon	Bamenda	HIV‐seropositive patients	135
(Antoinette and Dieudonne, 2021) [45]	May 2013–April 2014	Cameroon	Douala	Children	400
(Bikaula Ngwidiwo et al., 2021) [46]	2011–2014	RDC	Kinshasa	Everyone	8926
(Molamba et al., 2021) [47]	January 2010–September 2019	RDC	Kinshasa, Kongo Central	Patients with urinary calculi	194
(Ndzime et al., 2021) [48]	January 2018–June 2019	Gabon	Franceville	Everyone	1086
(Loumingou and Sinomono, 2021) [49]	January 2013–December 2015	Republic of Congo	Brazzaville	Adult patients	681
(Ngong et al., 2021) [50]	August–November 2017	Cameroon	Buea	Pregnant women	287
(Charlotte et al., 2022) [51]	January 2019–December 2021	RDC	Kinshasa	Diabetic patients	320
(Ikobo et al., 2022) [52]	June–November 2020	Republic of Congo	Brazzaville	Infants	201
(Ndzime et al., 2023) [53]	January 2018–December 2021	Gabon	Franceville	Children	508
(Mukubwa et al., 2023) [54]	June 2017–May 2018	RDC	Kinshasa	Everyone	2765
(Mourembou et al., 2024) [55]	1995–2015	Gabon	Libreville	Everyone	1262

### 3.2. Assessment of Methodological Biases Using the JBI (Joanna Briggs Institute) Tool

A formal assessment of the quality of the studies was carried out using the JBI critical appraisal tool, but this step was not detailed in the initial version of the manuscript. We have now added a full description of this assessment in the Methods section, and the corresponding results are shown in Table 2.

**Table 2:** Assessment of methodological biases according to the JBI tool.

**Quality**	**Score**	**Number of studies (%)**
Good	7–9	18 (59.94)
Moderate	4–6	14 (41.18)
Low	0–3	2 (5.88)

Evaluation according to the JBI criteria showed that, of the 34 included studies, 94.12% (32/34) had fairly good methodological quality, while 5.88% (2/34) had low methodological quality.

### 3.3. Prevalence of UTIs

**Figure 2:**
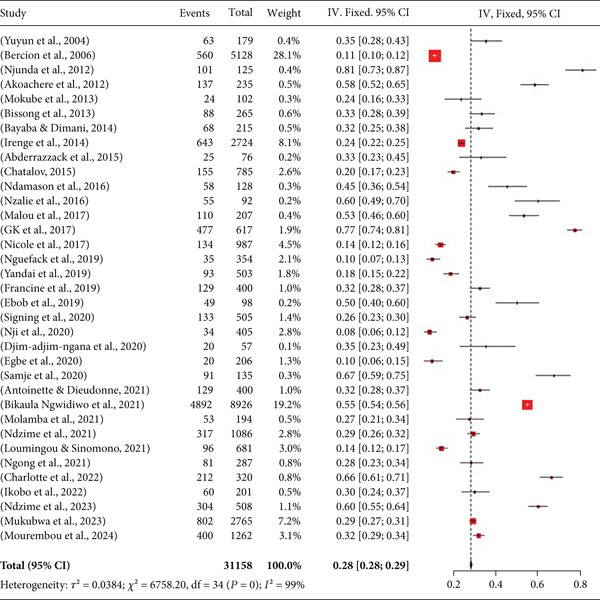
Meta‐analysis of urinary tract infections in Central Africa.

A meta‐analysis of the 34 articles on the prevalence of UTIs in Central Africa was carried out, and the data are presented in Figure 2.

The analysis in Figure 2 presents data with high heterogeneity (*I* ^2^ = 99) and *p* value = 0. Furthermore, the overall prevalence of UTIs in this analysis is 28%, with a 95% confidence interval between [28, 29]. This analysis also reveals that the prevalence of UTIs in Central Africa varies from study to study. The lowest recorded prevalence was 8% and the highest 81%, both obtained in Cameroon.

### 3.4. Assessment of Publication Bias

**Figure 3:**
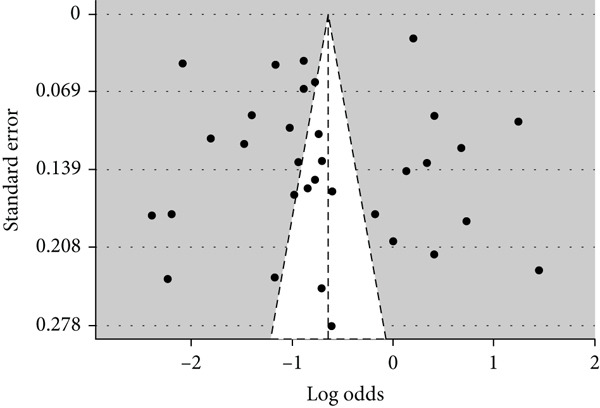
Visual analysis of publication bias via the funnel diagram.

**Figure 4:**
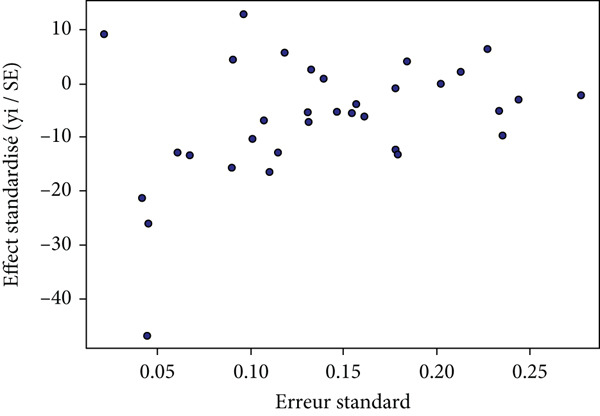
Egger′s test result for the evaluation of publication bias.

Publication bias was explored using the funnel diagram (Figure 3) and the Egger regression test (Figure 4).

The funnel diagram was used to visually assess the presence of publication bias. The overall distribution of studies appears to be relatively symmetrical (Figure 3), suggesting that there is no obvious publication bias. However, this visual assessment was supplemented by the Egger regression test (Figure 4).

Egger regression test results indicate that there is no statistically significant evidence of asymmetry (*t* = −1.16; ddl = 32; *p* = 0.2536). In addition, the estimation of the slope when the standard error approaches zero (*b* = −0.3009; 95% CI: −0.7687 to 0.1668) includes the value of zero, confirming the absence of detectable publication bias.

### 3.5. Prevalence of UTIs by Gender

**Figure 5:**
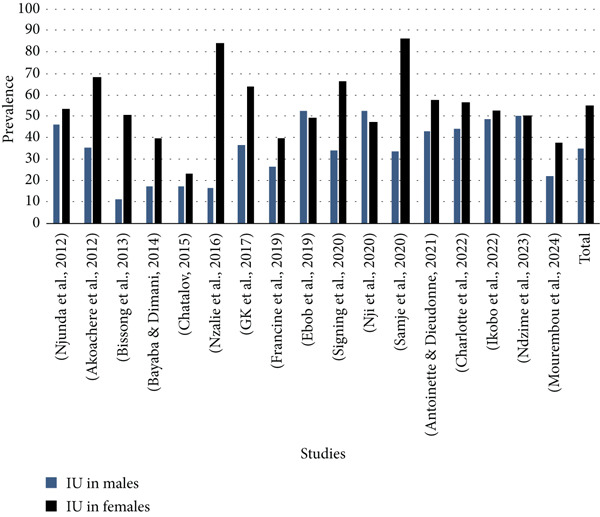
Prevalence of urinary tract infections by gender.

Analysis of the selected articles highlights that 50% (17/34) of the studies report the prevalence of UTIs in both males and females. These data are illustrated in Figure 5.

Analysis of Figure 5 suggests that in Central Africa as a whole, UTIs affect females more than males, with respective prevalences of 54.25% in females and 34.45% in males. The results show that 82.30% of the studies (14/17) underline that the prevalence of UTIs in females is higher than in males, whereas only 11.80% of the studies (2/17) record a higher prevalence in males. Finally, 5.90% of the studies (1/17) show equal prevalence between the sexes.

### 3.6. Isolation Rate of *Klebsiella* spp. in UTIs

**Figure 6:**
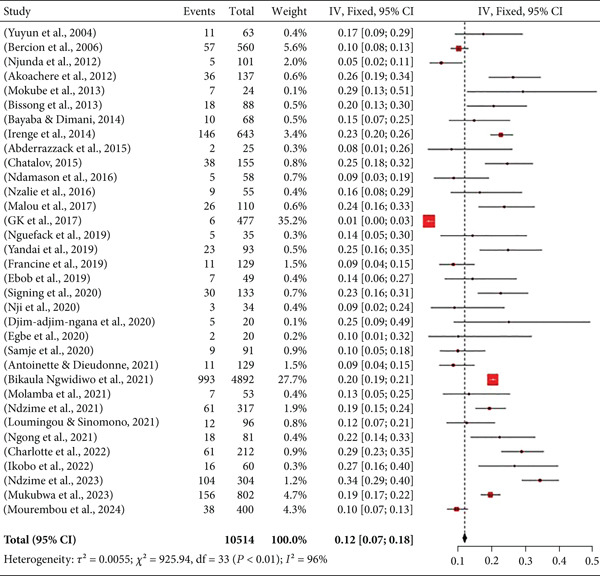
Meta‐analysis of the isolation rate of *Klebsiella* spp. in urinary tract infections in Central Africa.

A meta‐analysis was carried out on the selected studies, highlighting the rate of isolation of *Klebsiella* spp. responsible for UTIs in Central African countries (Figure 6).

The meta‐analysis of *Klebsiella* spp. isolation rates in UTIs in Central Africa reveals considerable heterogeneity in the data, with *I* ^²^ = 96*%*. Also, this analysis shows *Klebsiella* spp. isolation rates ranging from 1% to 34%. The overall isolation rate is 12%, with a 95% confidence interval of 7%–18%.

### 3.7. Distribution of *Klebsiella* spp. Isolation Rates in Central Africa

**Figure 7:**
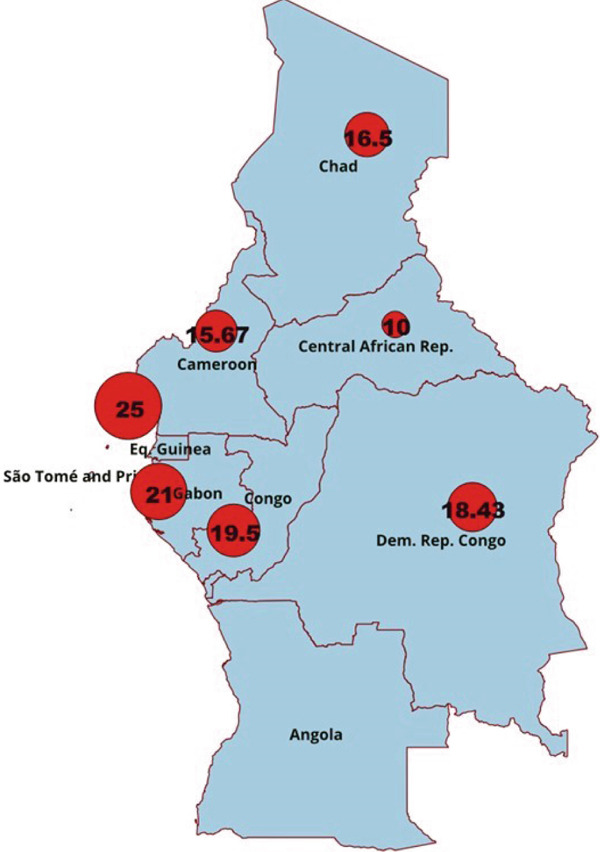
Distribution of *Klebsiella* spp. isolation rates in Central Africa.

Isolation rates of *Klebsiella* spp. in UTIs in Central Africa vary depending on the study and country. Figure 7 shows the distribution of *Klebsiella* spp. isolation rates by country in this region.

Analysis of the distribution of *Klebsiella* spp. isolation rates in UTIs in Central Africa shows that overall, *Klebsiella* spp. isolation varies from 10% to 25%. Figure 5 shows that the highest rates were recorded in Equatorial Guinea (25%), Gabon (21%), Congo (19.50%), and DRC, respectively. The average isolation rate was recorded in Cameroon (15.67). The lowest rate was obtained in the Central African Republic (10%).

### 3.8. Distribution of *Klebsiella* spp. Identification Methods and Tools

Several diagnostic methods, both manual and automated, were used to identify *Klebsiella* spp. isolates (Table 3). The results showed that manual methods (88.20%) were the most commonly used, followed by semiautomated methods (11.8%) and automated methods (0%). Among the manual methods used in Central Africa, there was a predominance of the API gallery (64.70%), followed by conventional culture methods (11.80%) and the Enterosystem 18R (5.90%).

**Table 3:** Distribution of *Klebsiella* identification methods and tools used in the included studies.

**Methods**	**Tools**	**Effectifs (%)**
Manuals	Enterosystem 18R	2 (5.90)
	API Gallery	22 (64.70)
	Cultivation methods	4 (11.80)
	RapID identification system	1 (2.90)
	Manual biochemical tests	1 (2.90)
Global manual		30 (88.20)
Semiautomated	Biochemical automata (VITEK 2)	4 (11.80)
Automated	MALDI‐TOF MS	0 (0.00)
	Molecular methods (PCR, sequencing)	0 (0.00)

### 3.9. Temporal Evolution of the Isolation Rate of *Klebsiella* spp.: Meta‐Regression

**Figure 8:**
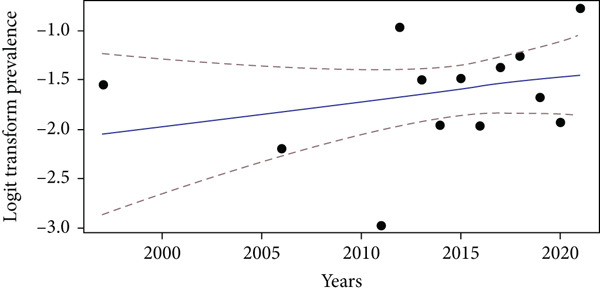
Mixed effects meta‐regression of the isolation rate of *Klebsiella* spp. according to the year. In blue: the estimated trend of prevalence based on years; in red: 95% confidence interval.

A meta‐regression was performed to evaluate the evolution of the isolation rate of *Klebsiella* spp. over time in urinary infections reported in Central Africa. Figure 8 below presents the regression curve.

Mixed‐effects meta‐regression (*k* = 13) shows a slight nonsignificant trend towards an increase in the isolation rate of *Klebsiella* over the years (coef = 0.025, *p* = 0.28). The heterogeneity between the studies remains very high (*I* ^2^ = 94.4*%*), suggesting that other factors explain the variability of the results. However, due to the lack of detailed data on subgroups in the studies conducted in Central Africa, it was not possible to further explore the sources of this heterogeneity through subgroup analyses or multivariate meta‐regression.

### 3.10. *Klebsiella* spp. Distribution by Species

**Figure 9:**
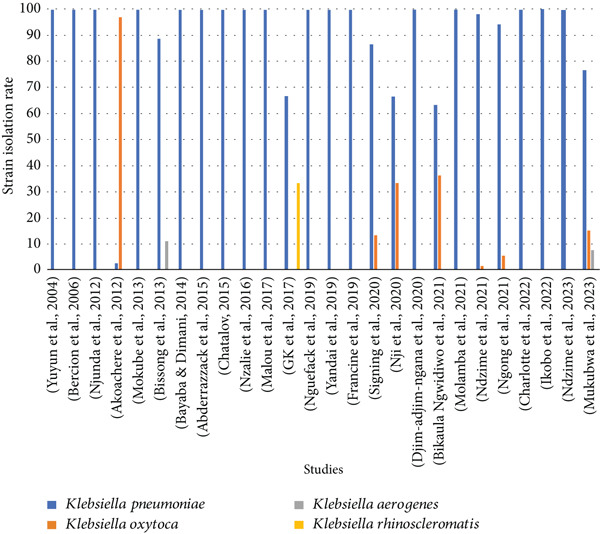
Distribution of different *Klebsiella* spp. species according to studies.

Of the studies selected, only 76.47% (26/34) specified the detection rate of *Klebsiella* bacteria in UTIs. These data are shown in Figure 9.

Figure 9 shows the four *Klebsiella* species most frequently isolated from UTIs in Central Africa. They are as follows: *Klebsiella pneumoniae*, *Klebsiella oxytoca*, *Klebsiella aerogenes*, and *Klebsiella rhinoscleromatis*. Moreover, *Klebsiella pneumoniae* was the species found in all studies 100% (26/26), followed by *Klebsiella oxytoca*, which was detected in 26.92% (7/26) of studies, while *Klebsiella aerogenes* and *Klebsiella rhinoscleromatis* were characterized in 7.69% (2/26) and 3.85% (1/26) of studies, respectively. *Klebsiella pneumoniae* was the bacterium with the highest isolation rate in 96.15% (25/26) of studies. Finally, among these studies, 3.85% (1/26) showed the simultaneous detection of 3 different species, and 30.77% (8/26) detected two species, compared with 65.28% (17/26) which showed only one species.

### 3.11. *Klebsiella* spp. Isolation Rates by Gender

Data on the distribution of the *Klebsiella* bacteria genus responsible for UTIs by gender is available in only three (3) of the 34 studies selected.

**Figure 10:**
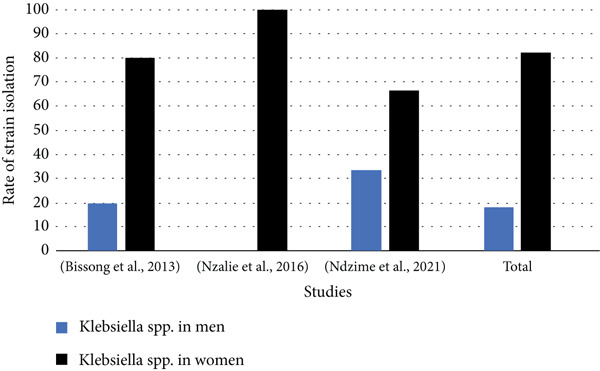
Distribution of *Klebsiella* spp. strains responsible for urinary tract infections according to gender.

Analysis of Figure 10 shows that *Klebsiella* spp. strains responsible for UTIs in Central Africa are predominantly identified in females, with overall isolation rates of 82.23% versus 17.77% in men.

These results highlight a significant burden of UTIs in Central Africa, with a substantial contribution from *Klebsiella* spp., underscoring the need to strengthen microbiological surveillance and implement appropriate management strategies.

## 4. Discussion

The main aim of this study was to determine the overall prevalence of UTIs in Central Africa and to provide additional data on the epidemiology of the *Klebsiella* bacteria responsible for these infections in this region. Data from studies of UTIs in Central Africa show that the highest prevalence was 81% [24] and the lowest 8% [41]. Both prevalences were obtained in studies conducted in Cameroon. This difference in prevalences seems to be explained by the type of study populations. Indeed, these prevalences were obtained, respectively, in diabetic patients and in children aged 0–3 years. Diabetes is recognized as a risk factor for UTIs, which would clearly explain this high prevalence [56]. Children, on the other hand, are in most cases well cared for by their parents in terms of hygiene and are therefore less exposed to UTIs. This analysis reveals that the overall prevalence of UTIs in Central Africa is 28%. This result is similar to that obtained in a meta‐analysis of UTIs worldwide by Salarie et al., in which the prevalence of UTIs was 23.90% [57]. However, this result is still higher than the prevalence obtained in a worldwide meta‐analysis of UTIs, which was 16% [10]. The difference in prevalence between these studies can be explained not only by population size but also by population type. Certainly, the study by Mengistu et al. covers the majority of populations in developed countries, where the health system is well developed and better organized, and therefore, the management of UTIs is better. As a result, these populations are less prone to UTIs than populations in Central Africa, where many populations lack access to quality healthcare and adequate treatment. In many studies, such as ours, focusing on UTIs, the prevalence of UTIs differs based on sex. The meta‐analysis of the data gathered in the present study also shows that the prevalence of UTIs is higher in women than in men in Central Africa. These results are confirmed by several studies [58, 59]. However, this difference can be explained by anatomical–physiological differences between the sexes. Indeed, the shorter urethral canal in women and the proximity of the urinary meatus to the anus in the latter favor an increase in UTIs. Physiological conditions such as pregnancy or menopause also predispose women to UTIs [60].

Many pathogens are implicated in these infections, including *Klebsiella* spp. In this study, *Klebsiella* spp. isolation rates ranged from 1% [35] to 34% [53]. The major difference in the rates of *Klebsiella* spp. isolation between these two studies can be explained by the methods used to identify the strains. Ndzime et al. [53] used the VITEK‐2 system from bio‐Mérieux, one of the current reference methods for bacteriological identification. In contrast, Bunduki et al. [35] used conventional biochemical tests, which are prone to false positive outcomes due to the biochemical similarity of many bacteria, but also to the loss of the ability to hydrolyze substrates. Nevertheless, the overall isolation rate of *Klebsiella* spp. in the present study was 12%. This result is similar to that obtained in a study of UTIs in developing countries in Africa and Asia, in which the *Klebsiella* spp. isolation rate was 14.60% [61]. Our reported isolation rate of *Klebsiella* spp. in Central Africa remains far higher than the rates reported by Maldonado Barragán et al. on UTIs in symptomatic patients in East Africa and De Souza et al. on pregnant women from Latin America, where *Klebsiella* spp. were isolated at respective rates of 5.80% and 6.40% [14, 58]. This large difference in the rates of isolation between our study and those conducted in different regions of Africa and Latin America reveals that Central Africa is a region of great public health concern. It is suspected that this pathogen is constantly evolving epidemiologically, in terms of its virulence and the mortality rate of the infected patients. These large differences also highlight the inadequacy of urinary tract diagnosis tools in these countries. Indeed, in the studies reviewed in Central Africa, all diagnosis methods were based on bacterial cultures and biochemical identifications of uropathogens from urine samples. Although these are standard methods for diagnosing UTIs, they remain prone to errors and outcome misinterpretations. This argument is supported by a study of Van Der Zee et al. [62] which suggests that 25%–30% of urine cultures produced negative results in symptomatic patients. Moreover, in some polymicrobial infections, one organism may have a higher growth rate than the others, thus allowing its identification alone at the expense of the others. For instance, *Escherichia coli*, which grows rapidly in standard culture media, can competitively suppress other uropathogens [63]. These scenarios highlight the need to develop more accurate, specific, and faster diagnostic methods for identifying pathogens involved in UTIs. Recently, several pioneering studies have suggested that there could be substantial improvements in the identification of polymicrobial pathogens using molecular tests based on polymerase chain reaction (PCR) technology [63, 64]. The work of Kapoor et al. reported that a PCR‐based method was positive for uropathogen identifications in 35% of previously defined sterile urine by bacterial culture methods [63].

The frequency of isolation of *Klebsiella* spp. in UTIs in Central Africa indicates that the highest rate of isolation was obtained in Equatorial Guinea. This result could be explained by the fact that only one study was carried out in that country and taken into account for the isolation rate of *Klebsiella* spp. The same is true for the Central African Republic, which has a 10% isolation rate. Nevertheless, our results show that isolation rates in countries such as Cameroon are 15.67% (18 studies) and the Democratic Republic of the Congo 18.40% (seven studies) do not depart significantly from the overall isolation rate of *Klebsiella* spp. in Central Africa reported in the present study.

*Klebsiella pneumoniae* is the most represented bacterial species in this study. This result is similar to those obtained in many other studies worldwide [65, 66]. Indeed, *Klebsiella pneumoniae* is widespread in human clinical collections, representing an isolation rate of about 85% [67]. This high rate of isolation makes this species the most dominant and studied from this genus. However, the low isolation rate of *Klebsiella aerogenes* in UTIs in Central Africa could be justified by the fact that this species was long wrongly classified in the genus *Enterobacter* spp. [68]. This confusion stems from the similarity in crop parameters and biochemical identifications of the genus *Enterobacter* which had long been misidentified as *Enterobacter aerogenes*. This bacterium is now undergoing a taxonomic re‐evaluation. Although reclassified as *Klebsiella aerogenes*, hospital staff, clinicians, and researchers are still unable to assimilate this taxonomic change due to a lack of scientific rigor. In addition, our study reveals that *Klebsiella rhinoscleromatis* was only isolated in one study. This very interesting result would be due to the complexity of the identification of this bacterial species. This species has many similarities with *Klebsiella pneumoniae*, which makes its identification based on biochemical properties difficult [69]. *Klebsiella rhinoscleromatis* is recognized as the causative agent of rhino‐sclerosis (chronic granulomatous infection of the upper respiratory tract), unlike *Klebsiella pneumoniae*, which can be found in many hosts, environments, and clinical sources. Although identified as the causative agent of rhinosclerosis, it is often identified in urinary infections [70]. The identification methods used for the detection of uropathogens varied widely between studies, reflecting significant disparities in the technical capacities and resources of laboratories in the region. Manual methods accounted for the majority of approaches (88.2%), with a predominance of the API gallery. Although this method is widely used in routine practice in many resource‐limited countries, it has limitations in terms of accuracy, particularly in differentiating closely related species within the *Klebsiella* genus.

The limited use of semiautomated tools (such as VITEK 2) and the complete absence of modern methods such as MALDI‐TOF MS or PCR in the included studies highlight a major technological constraint. This may have led to errors or inaccuracies in species identification, potentially influencing the results. Consequently, the assessment of species diversity, as well as dissemination mechanisms specific to each species, remains only partially explored. This gap highlights the need to improve diagnostic techniques and methodological rigor in future studies in order to obtain more complete and reliable data, which are essential to guide clinical management strategies and epidemiological surveillance.

The strains of *Klebsiella* spp. responsible for UTIs in Central Africa are mostly identified in females than in males, although the relationship between the dynamics of urinary microbiota composition and the prevalence of UTIs is not yet clearly understood. However, the predominance of *Klebsiella* spp. bacteria in UTIs in women compared to men is explained by the imbalance of the urinary microbiota. The female urinary meatus is in close contact with the vaginal apparatus, which is a source of several imbalances causing the proliferation of pathogenic microorganisms, which will in many cases go up by inappropriate gestures to the urinary meatus [71]. These bacteria are known as natural protective barriers against UTIs. Nevertheless, women are more prone to urogenital microbiota imbalances due to lack of natural or associated hygiene with estrogen treatments, birth control pills, and bacterial vaginosis [72].

There are some limitations to this review. First, the heterogeneity between the included studies was very high, which affects the precision of the pooled estimates. Second, the limited number of studies available in Central Africa and the lack of data by age, sex, and many other variables have restricted the possibility of performing subgroup analyses or multivariate meta‐regressions. In addition, accurate identification of *Klebsiella* species was performed in only a portion of the studies, limiting the analysis of bacterial diversity. Despite these limitations, this study provides one of the most comprehensive summaries to date on *Klebsiella* spp. UTIs in Central Africa.

## 5. Conclusion

The present study provided epidemiological data on UTIs caused by bacteria of the genus *Klebsiella* spp. in Central Africa. The overall pooled prevalence of UTIs in Central Africa was estimated at 28%. The overall isolation rate of *Klebsiella* spp. involved in these UTIs was 12%. This rate varied not only between countries in the subregion, but also between species. *Klebsiella pneumoniae* remains the most prevalent species among the bacteria of the genus *Klebsiella* spp. involved in UTIs in Central Africa in line with world trends. These data highlight not only the need to measure the impact of these infections on populations but also the need to develop or adopt more effective diagnostic tools for these uropathogens in Central African countries in order to obtain more reliable results on the identification of pathogens. This will require the implementation of monitoring programs and require additional studies focusing on the antibiotic resistance profiles and clonal diversity of these bacteria in order to evaluate their degree of dissemination in the region.

## Conflicts of Interest

The authors declare no conflicts of interest.

## Author Contributions

**E.M.N.:** conceptualization, data curation, investigation, visualization, writing—original draft, and writing—review and editing. **F.M.:** data curation, formal analysis, and validation. **M.M.:** methodology and data curation. **R.M.M.:** validation, writing—original draft, and writing—review and editing. **O.Z.M.:** investigation, methodology, and visualization. **L.F.O.:** methodology and data curation. **J.F.Y.:** conceptualization, resources, supervision, and writing—review and editing.

## Funding

No funding was received for this manuscript.

## Data Availability

The data that support the findings of this study are openly available in PROSPERO at https://www.crd.york.ac.uk/PROSPERO/view/CRD42024572022, reference number CRD42024572022.
